# Aloe vera vs chlorhexidine in preventing alveolar osteitis

**DOI:** 10.6026/973206300200993

**Published:** 2024-09-30

**Authors:** Shruti Ajmera, Sunil Sharma, Amit Kumar Sharma, Vikram Sharma, S Meera Petchiammal, Shraddha Sinha

**Affiliations:** 1 Department of oral and maxillofacial surgery, Nims dental college and Hospital, Nims University, Jaipur, India

**Keywords:** Aloe vera, chlorhexidine, dry socket, third molar surgery, alveolar osteitis, mouthwash

## Abstract

Alveolar Osteitis (AO) is a common and painful complication following tooth extraction or surgical excision, necessitating early treatment to minimize costs, morbidity, and frequent dental visits. Chlorhexidine (CHX) is the most widely used antiseptic, while Aloe Vera, a natural herbal plant, is gaining popularity in dentistry due to its minimal side effects. This study aimed to evaluate the efficacy of normal saline, Aloe Vera, and chlorhexidine mouthwashes in reducing the incidence of AO after lower third molar surgery. In a randomized controlled trial, 150 patients were divided into three groups: Group 1 (Chlorhexidine Mouthwash), Group 2 (Aloe Vera Mouthwash), and Group 3 (Normal Saline, placebo). Postoperative assessments on the 2nd and 7th days measured Trismus Grading, VAS, Wound Dehiscence, and AO. The results showed no significant difference in the occurrence of AO and Trismus grading between groups (p = 0.031 and 0.78, respectively), but wound dehiscence significantly differed (p = 0.013). While the VAS score on the 2nd day showed no significant difference, by the 7th day, chlorhexidine demonstrated better pain reduction than Aloe Vera. Although both mouthwashes were effective in reducing AO, chlorhexidine was more effective in reducing wound dehiscence and pain by the 7th day post-surgery.

## Background:

One well-known side effect following tooth extraction or surgical excision is Alveolar Osteitis (AO). Often referred to as "dry socket," this issue is still a frequent surgical aftereffect that causes excruciating pain and need frequent follow-up appointments [1, 2]. Recently, AO is described as "pain postoperatively inside and around the extraction socket, with or without halitosis, accompanied by a partial or complete disintegrated blood clot within the alveolar socket that hikes in severity at any time from first and third day after the extraction" [3]. Between 1% and 37.5% of people experience AO after the extraction of their lower third molars [4]. Because Chlorhexidine gel has broad spectrum activity, covers anaerobes, and has no registered resistance, some research has looked at how it can prevent AO [5]. The most widely used medicinal plant in the world, Aloe Vera is the oldest known herbal remedy. Although the aloe plant has more than 300 species, the Aloe barbadensis species has the most beneficial medical qualities [6]. Aloe Vera possesses a number of significant medicinal qualities, including as immunomodulation, moisturizing and anti-aging, antiviral and antitumor activity, healing, heat injury healing, and anti-inflammation [7, 8-9]. Aloe Vera, a natural herbal plant, has gained popularity in dentistry and medicine due to its benefits, including increasing granulation tissue's collagen content and degree of crosslinking by lowering acid solubility and increasing aldehyde content, without known negative effects. As dentists, we face the challenge of recommending treatments while being mindful of potential side effects. This study aims to evaluate the efficacy of traditional normal saline mouthwash rinses, Aloe Vera, and chlorhexidine in reducing the frequency of alveolar osteitis following surgical lower third molar procedures. Objectives include determining and assessing the prevalence of alveolar osteitis, evaluating postoperative wound dehiscence, measuring postoperative pain using the VAS scale, and assessing the grade of postoperative trismus.

## Material and Methods:

The study was approved by the institutional ethics board - IEC/P-08/2022. Prior to commencement, written consent was acquired from the Institutional Ethics Committee. Additionally, written approval was obtained from the oral and maxillofacial surgery department and other relevant departments. Patients who met exclusion and criteria and who got admitted in oral and maxillofacial surgery ward of NIMS Dental College and Hospital during the study period were included after obtaining verbal consent with full information provided.

## Inclusion Criteria:

Age group of patients (Male or Female) 30 years old and above, Patient who agreed to follow the study protocol, Patient who undergoes extraction of mandibular third molar surgery, Patient who underwent extraction from the department of oral surgery, at tertiary care center.

## Exclusion criteria:

Pre-existing infections, Patients having primary and mixed dentition, Systemically compromised patients, uncontrolled hypertension, diabetes mellitus, Immune suppression, bone pathology, Pregnancy or lactation, females taking oral contraceptives, Allergy to chlorhexidine and Aloe Vera mouthwash, Chain smokers, Patients with a previous history of difficult extraction and those with any neurological deficits. A detailed case history was recorded in a specially designed proforma included demographic details, contact information, past medical history, habits, oral hygiene habits, intraoral examination before and post operatively by using trismus score, VAS, wound dehiscence score. A pre-designed and pre-tested questionnaire was employed to collect the required information. A simple random sampling technique was used to allocate the patients in three groups. A comparative study was done on 150 patients clinically diagnosed with mandibular impacted 3rd molars.

## Clinical re-evaluation:

On the second postoperative day, reassess the patients. On the seventh postoperative day, carry out another assessment.

## Procedure:

The procedure was carried out by one operator under strict aseptic precautions. Standard protocols were followed for the draping processes and povidine iodine was used in the standard painting and cleaning procedures. In order to induce anesthesia, lingual and buccal nerve blocks, as well as intraoral inferior alveolar nerve blocks, were performed using 2% lignocaine HCl containing 1:200,000 adrenaline. Patients in all three groups were given information on how to rinse after surgery according to the approved regimen. Group C was given regular saline, whereas group A, B received 150 cc bottles of Aloe Vera and 0.2% chlorhexidine mouthwash, respectively.

## Postoperative Instructions:

## Regular post-operative instructions were given to all the patients along with these medications:

[1] Paracetamol 650 mg one tab four to six hourly

[2] Amoxycillin 500 mg with clavulanate potassium 125 mg eight hourly for 5 days.

## Rinsing protocol:

Following surgery, mouthwash should be rinsed twice daily in accordance with the following instructions: Daily limit of 10 cc of mouthwash will be given, Patient's needs to use the mouthwash for a 30-second, Patients will rinse their mouths completely with clean water after spitting out the mouthwash, After using the mouthwash, patients should wait at least 30 minutes before eating anything.

## Follow-up and observation:

Every patient was evaluated at two distinct intervals:- Two days after surgery; Seven days after surgery. The patients rated the intensity of their pain from 1-10 with the help of Visual Analogue scale (VAS). Post-operatively wound dehiscence, Alveolar Osteitis and Trismus were assessed clinically.

## Results:

The present comparative study conducted in Department of oral and maxillofacial surgery at a tertiary care centre during study period 1.5 years (June 2022 to December 2023) all 150 patients undergoing mandibular third molar surgery, according to exclusion and inclusion criteria admitted to oral and maxillofacial surgery ward of tertiary care centre such cases were included in the study. Distribution of cases among male and female in three different groups out of 50 patients is done. Male and female cases are 33 and 17 in Chlorhexidine group, In Group 2 male and female cases are 37 and 13 respectively and in group 3(the control group) male and female cases are 38 and 12 respectively (Table 1). Percentage of Alveolar osteitis in three different groups respectively, percentage of post-operative wound dehiscence and percentage of postoperative trismus grading along with p value for each group respectively. Occurance of alveolar osteitis and Trismus grading is statistically not significant in three groups as p value is 0.031and 0.78 respectively. However, p value is 0.013 in post-operative wound dehiscence (Table 2).VAS score on post-operative day 2 and day 7 in three different groups respectively. Day 2 shows no significant comparison. However there is a significant comparison on day 7 between three different groups (Table 3).

## Discussion:

In oral and maxillofacial surgery, lower third molar impactions come under everyday minor oral surgical procedures. The most recurrent postoperative consequence is the development of Alveolar Osteitis or localized osteitis. [11] Present study examines the effects of three different mouthwash rinses after surgery on trismus, discomfort, wound dehiscence distal to the second molar, and the occurrence of alveolar osteitis following surgery. In total, 150 patients were involved in the research. Group A reported less number of wound dehiscence and a low pain score during the seven-day follow-up. It was suggested that fibrinolysis and bacterial infection combine to induce AO. It might be stopped by lowering the number of microbes in and around the surgical site [12]. Chlorhexidine (CHX) works against viruses by altering cell membrane permeability, which helps it to deactivate enveloped viruses like herpes simplex virus, known for causing cold sores. However, CHX shows limited effectiveness against non-enveloped viruses, such as human papilloma virus (HPV), which can be associated with oral cancers [13,14,15]. At this dosage, Chlorhexidine has bactericidal effects by rupturing the integrity of bacterial cell membranes and changing the osmotic balance in bacteria [16]. In a study by Larsen (1991)[17], researchers examined the effectiveness of using a 0.12% chlorhexidine mouthwash in preventing Alveolar Osteitis (AO) following the surgical removal of 85 mandibular third molars. Their findings revealed a significant reduction in AO prevalence, with a 60% decrease compared to the control group. Similarly, Hermesch *et al.* (1998) [18] conducted a study investigating the impact of a 0.12% chlorhexidine mouthwash on AO occurrence following the excision of impacted mandibular third molars. Their results showed a notable 38% reduction in AO occurrence compared to the control group [18]. In present study, Chlorhexidine mouthwash rinses were clinically better than Aloe Vera mouthwash and control group in preventing Alveolar Osteitis but statistically not significant. The study conducted by Kathuria *et al.* underscores the multifaceted benefits of Aloe Vera in oral healthcare. Aloe Vera's antibacterial properties which prove effective in combating various oral ailments, including bad breath, gingivitis, stomatitis, and periodontitis is known [19]. In this study, A. vera was administered as a mouthwash and the control group received saline mouthwash as a placebo. Analysis of facial swelling revealed a consistent decrease in swelling percentages in the A. vera group compared to the control group, with statistically significant differences observed on day 3 post-surgery. Additionally, the A. vera group experienced significantly lower pain intensity on days 1 and 3, indicating its potential in alleviating postoperative discomfort. Present study findings also indicated that rinses with Aloe Vera could lessen discomfort, reduce pain and trismus. In summary, A. Vera mouthwash lowers surgical complications following surgery, especially pain and swelling. Postoperative pain during day 2 was not significant but on day 7 it is significant compared to the control group. In a study by Mariotti and Rumpf [20], the impact of CHX on collagen synthesis was investigated. The researchers speculated that at concentrations that don't significantly affect cellular growth, CHX could greatly reduce the production of both collagen and non-collagen proteins involved in wound healing. The frequency of follow-ups and more sample size could add advantage to the present study to study the effectiveness of Three Groups in wound healing. Routine rinses with Chlorhexidine mouthwash and Aloe Vera mouthwash can be an efficient postoperative adjunctive treatment option for preventing occurrence of Alveolar Osteitis. Nevertheless, Chlorhexidine proved to have more potential to prevent wound dehiscence and pain in subjects as compared to rinses with aloe vera mouthwash.

## Conclusion:

Present Study concludes, rinsing with Chlorhexidine mouthwash is more efficient than Aloe Vera mouthwash in reducing occurrence of wound dehiscence and pain after 7 days of third molar surgery. Chlorhexidine mouthwash is more efficient in reducing the occurrence of Alveolar Osteitis than Aloe Vera mouthwash and control group clinically. However, no significant difference in the efficacy of Chlorhexidine mouthwash and Aloe Vera mouthwash was found in incidence of alveolar osteitis.

## Figures and Tables

**Table 1:** Below table shows Distribution of cases among male and female in three different groups out of 50 patients

**Group**		**sex**	
		**Male**	**Female**
chlorhexidine	Count	33	17
	% within Group	66.00%	34.00%
Aloe vera	Count	37	13
	% within Group	74.00%	26.00%
control	Count	38	12
	% within Group	76.00%	24.00%

**Table 2:** Below table shows percentage of Alveolar osteitis in three different groups , percentage of post-operative wound dehiscence and percentage of postoperative trismus grading along with p value for each group respectively

		**Group**			
		**chx**	**Aloe vera**	**control**	**P value**
Alveolar osteitis	Absent (%)	45(90%)	44(88%)	40(80%)	0.31
	Present (%)	5(10%)	6(12%)	10(20%)	
Post - operative wound dehiscence	Absent (%)	49(98%)	47(94%)	41(82%)	0.013*
	Present (%)	1(2%)	3(6%)	9(18%)	
Trismus grade	0	36(72%)	32(64%)	30(60%)	0.78
	1	10(20%)	13(26%)	13(26%)	
	2	4(8%)	4(8%)	5(10%)	
	3	0(0%)	1(2%)	2(4%)	

**Table 3:** Below table shows VAS score on post operatively day 2 and day 7 in three different groups respectively. Day 2 shows no significant comparison. However there is a significant comparison on day 7 between three different groups

VAS	Group			P value
	chx		control	
VAS (Day 2)				0.65
Mild	37(35%)	35(%)	31(30.1%)	
Moderate	10(28.6%)	10(28.6%)	15(42.9%)	
Severe	3(25%)	5(41.7%)	4(33.3%)	
VAS (Day 7)				0.015*
Mild	46(39.3%)	39(33.3%)	32(27.4%)	
Moderate	13.8%)	10(34.5%)	15(51.7%)	
Severe	0(0%)	1(25%)	3(75%)	
